# A comparison of the parental values of children’s extracurricular music learning in Guilin, China and in Tampa, United States

**DOI:** 10.3389/fpsyg.2024.1275734

**Published:** 2024-01-30

**Authors:** Cancan Cui, Xin Xie

**Affiliations:** ^1^College of Music and Dance, Guangzhou University, Guangzhou, China; ^2^School of Music, The Pennsylvania State University, University Park, PA, United States

**Keywords:** parental values, cultural differences, extracurricular music learning, socioeconomic status (SES), background

## Abstract

Many parents enroll their children in extracurricular music activities. However, cultural differences may contribute to parents’ different values that ultimately influence their behaviors and attitudes. This cross-cultural study aimed to compare the value parents have on their children’s private music education by examining four cases in Guilin, Guangxi (China) and Tampa, Florida (United States). Two main research questions guided this study: (1) How do parents in Guilin and Tampa perceive and value their children’s music learning? (2) Does the value parents hold for their children’s extracurricular music activities differ across different cultural contexts? If so, how do parents’ values and perceptions of their children’s involvement in extracurricular music activities differ between the contexts of Guilin and Tampa? We purposefully selected participants from two regions based on these criteria: (a) the participants included a single-child family and a two-child family in each of the two regions; (b) children’s age ranged from 5 to 12 years and lived with their biological parents (c) children were currently taking music lessons after school. We interviewed both children and parents during the data collection. The key findings reveal that parental values transcend two cultural contexts, specifically in (1) motivations for music learning, (2) expectations of music learning, (3) utilizing personal background, and (4) strategies for success. The findings suggest that several factors, such as children’s autonomy, musical joy, pursuit of musical career, “beauty development” and achievements, may have contributed to parents’ values. The implication for parents, psychologists, and policymakers is to understand the diverse needs and values within different cultural backgrounds, to promote the children’s development, to design curricula, and to use effective teaching methods in music education. In conclusion, both Guilin’s and Tampa’s parents’ values varied due to cultural differences, which further influenced their behaviors, attitudes and perceptions toward their children’s musical experiences.

## Introduction

Cultural differences have been frequently emphasized in the realm of education, psychology, and social studies, but have rarely been examined through the lens of music and parenting. Existing evidence has shown the outstanding achievements of Chinese musicians to their parents, as their parents dedicate endless physical and personal support in fostering and cultivating their children in extracurricular music learning (Yang, 2009; Kong, 2018). Nevertheless, U.S. parents paid more attention on affective and cognitive support during with their children’s musical learning journey (Zdzinski, 1996). It is obvious that with discrepant cultures, parents possess diverse attitudes and behaviors toward their children’s musical experiences.

Parenting is a lifelong effort. Regardless of parents’ backgrounds, they want their children’s lives to go easy and well (Baker and Barg, 2019). With a purpose of satisfying parents’ anticipation of happiness, fulfilling of lives, and enjoy financial security for their children, they acquire basic ideas about their children’s personal growth and development (McPherson, 2009; Baker and Barg, 2019). In fact, parents expect far more beyond than worries about happiness, fulfilling lives, and financial security (Baker and Barg, 2019). As Baker and Barg stated, they desire their children to have “certain qualities.” In other words, they aimed to foster and shape their children to be the people they want them to be. That is “parental values.”

The term ‘values’ is used when discussing human behavior with regards to the two unique perspectives of economic and customer behavior which are subjective and personal in nature (Moore and Asay, 2012). Values also have a major influence on a person’s behavior and thoughts and serve as broad guideline in all situations (Moore and Asay, 2012). Parental values can motivate different patterns of behaviors due to their pivotal role in manifesting cultural differences between socially and economically diverse groups. Parental values can be defined as parents’ thoughts, behaviors, and attitudes about the nature of child development and growth, which further influence their judgment and decision making (Tulviste et al., 2012). These thoughts and behaviors evolve slowly over time as part of the individual’s social and psychological development, and as a central part of family life (Brighouse and Swift, 2014). This cross-cultural comparative paper provides a comprehensive analysis of parental values in children’s extracurricular music learning in Guilin, China and Tampa, United States.

### Rationale for the study

A comparative study involves the systematic investigation of cultures and practices in different countries and regions to explore their similarities and differences; this approach can be applied in the fields of education, psychology, and social study (Manzon, 2007; Johansen, 2013). Comparative analysis examines the major factors impacting education development in relation to regional contexts and compares similarities and differences among different countries or regions, sometimes focusing on time as a unit for comparison (Kong, 2023).

**Figure 1:**
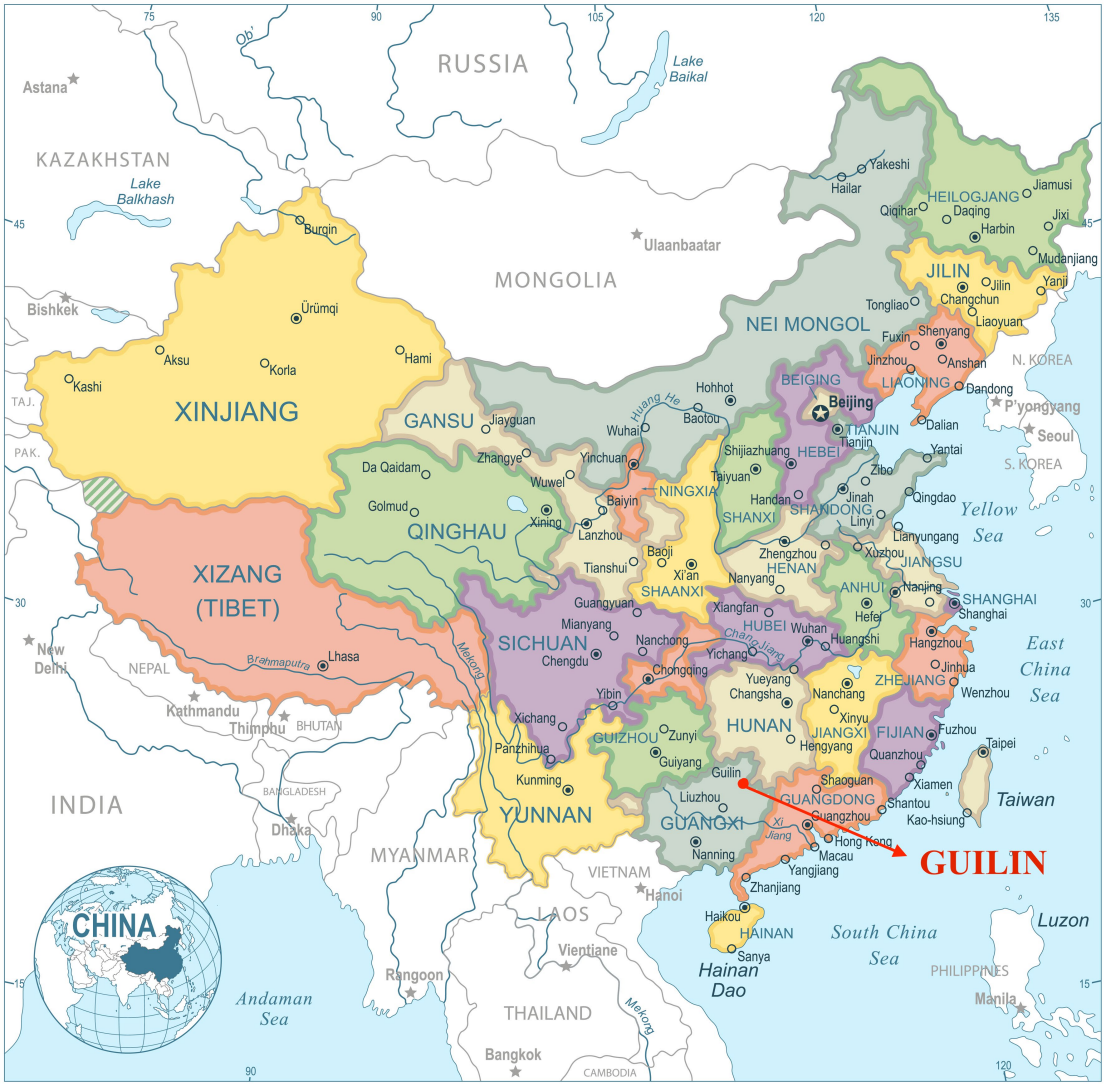
Location of Guilin. Image source: dikobrazik/DepositPhotos. Reproduced under the terms of DepositPhotos’ standard license.

**Figure 2:**
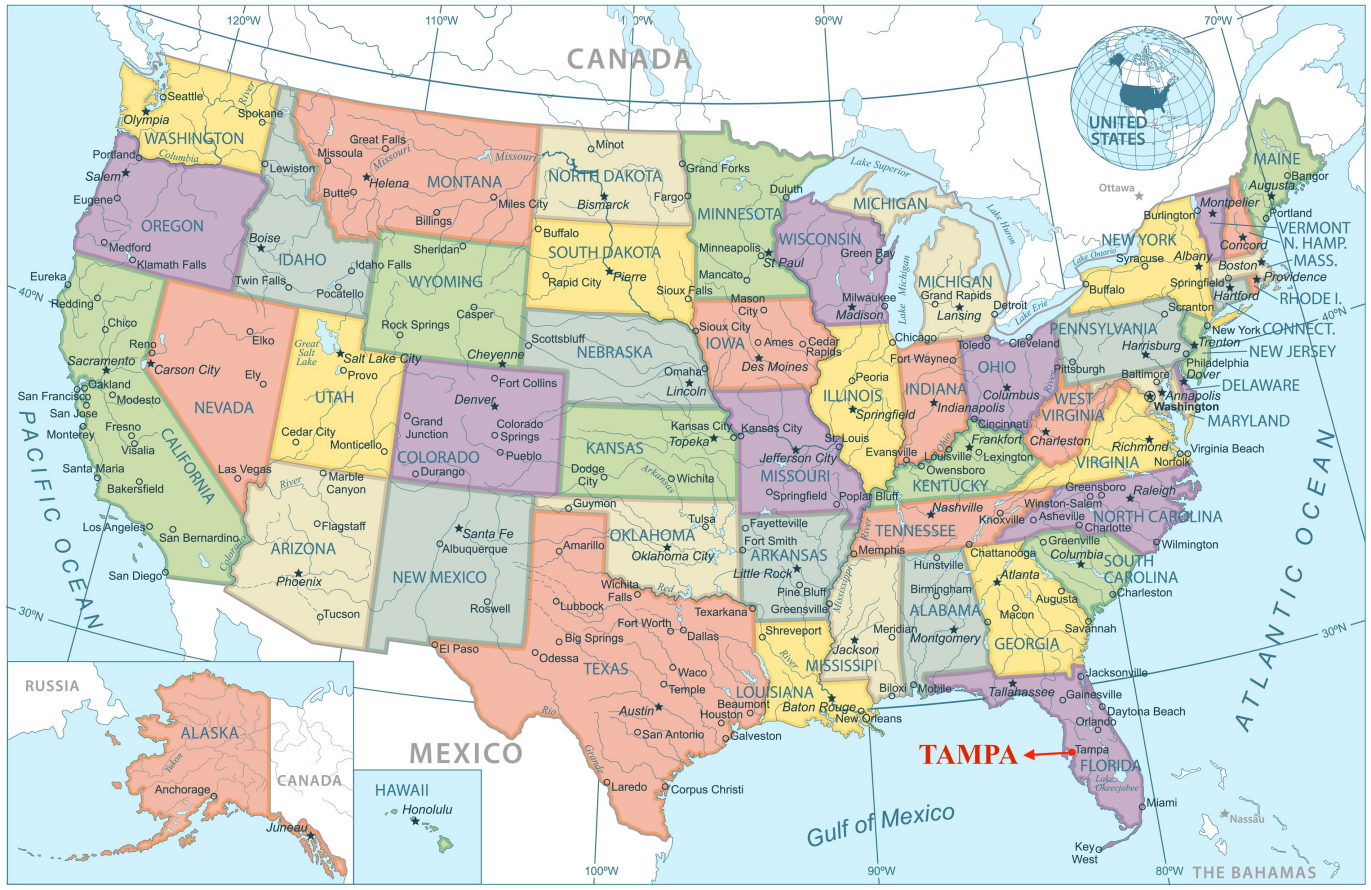
Location of Tampa. Image source: dikobrazik/DepositPhotos. Reproduced under the terms of DepositPhotos’ standard license.

In the current study, it is noteworthy that the two selected regions: Guilin, Guangxi, China and Tampa, Florida, United States, are comparable. First and foremost, both regions possess closed latitude in the south of each country (Tampa: 27.9506° N, 82.4572° W; Guilin: 25.2345° N, 110.1800° E) (see Figures 1, 2) and the latitudinal proximity indicates that a similar climate at further affects the people’s lifestyles and living patterns in each city. Second, both cities are of a similar size category, medium-sized on a global scale and are popular tourist destinations. Third, both cities are considered the medium size city in their own country. Fourth, the first author has lived in both cities and is familiar with the contexts of both cities. The abovementioned information strengthens the comparability of the two selected regions, which might also potentially influence the construction of parents’ values toward their children’s extracurricular music learning.

However, limited number of cross-cultural comparative research relevant to parenting through the lens of music has been conducted. In this way, conducting a cross-cultural study in which parental values relevant to music learning are compared in both Chinese and U.S. contexts is highly important. By identifying the similarities and differences between Guilin, China and Tampa, United States, researchers, psychologists, and educators might gain insights into improving the exchange of ideas among parents, children and educators from various countries, and the ongoing efforts may contribute to improving the effectiveness of future educators, psychologists, and administrators to be more effective.

## Literature review

### Socioeconomic status

Parental values differ based on social class, which influences children’s learning participation (Kohn, 1976; Lareau, 2000; Shih and Yi, 2014). Shih and Yi (2014) examined the values of parents of different socioeconomic status (SESs) and found that children with higher-SES parents were more likely to be involved in multiple activities and vice versa. Specifically, studies suggest that income, as one key dimension of parental SES, significantly influences parental values (McPherson, 2009; Elpus and Abril, 2011; National Center for Education Status, 2012; Shih and Yi, 2014; Singh, 2016). Middle-class families prioritize education more than lower-class and working-class families. For example, they recognize the positive impact of music learning on overall educational achievement (Ogbu, 1974; Lightfoot, 1978; Zdzinski, 2013; Shih and Yi, 2014; Dell et al., 2015; Barnes et al., 2016).

In contrast, working-class and low-income parents face challenges in providing support and managing their children’s activities due to limited resources and financial constraints (Hornby and Blackwell, 2018). One significant issue is their lower participation compared to parents from the middle-class brackets. Factors such as inflexible job schedules, a lack of childcare options, and transportation difficulties act as barriers that limit their involvement (Lee and Bowen, 2006; Hornby and Blackwell, 2018). Additional obstacles, as noted by Phillips (2003) and Caro (2018), include the high costs associated with registration fees, instrument rentals, and private music lessons, which are considered financial burdens that limit parents’ actions and beliefs. Paradoxically, this does not imply that low-income parents do not value music education (Jarrett and Coba-Rodriguez, 2015; Caro, 2018; Tan, 2019). Participants in Jarrett and Coba-Rodriguez’s (2015) study expressed unconditional support for their children’s pursuit of music as an extracurricular activity despite being from low-income families. In other words, parental values have no relationship with social class to some degree and, in turn, do not influence children’s learning participation (Jarrett and Coba-Rodriguez, 2015; Tan, 2019).

### Parental education

Research has established a connection between parents’ educational background and their parenting values (Baker and Stevenson, 1986; Shih and Yi, 2014; Hornby and Blackwell, 2018). Parents with higher education levels exhibit a greater desire to be involved in their children’s performance and have strong social connections with schools because they actively participate in school activities (Baker and Stevenson, 1986). Moreover, educated parents are more familiar with their children’s academic achievement, connect with their children’s teachers, and can evaluate their children’s academic performance (Baker and Stevenson, 1986; Hornby and Blackwell, 2018). These parents ensure attendance at parent-teacher meetings as well as children’s concerts and activities (Baker and Stevenson, 1986; Hornby and Blackwell, 2018). Additionally, highly educated parents have well-defined plans for their children (Baker and Stevenson, 1986).

In contrast, recent research has indicated that parental educational background does not necessarily determine parenting values (Shih and Yi, 2014). Some parents with lower education levels, even those whose children have lower academic performance, actively participate in their children’s learning (Hornby and Blackwell, 2018). Thus, parenting education background has no influence in parenting values.

### Parents’ music experiences

In addition to parents’ SES and parent’s education background, parents’ musical experiences indirectly influence to parenting value, especially in the context of music education (Margiotta, 2011; Ekong, 2013; Suk, 2014). Researchers have found that parents who have music or art backgrounds are more supportive of children’s music education journeys due to their own interests (Ho, 2011; Ekong, 2013). In addition, parents with musical backgrounds supervise and participate in their children’s home music practice closely more than parents without musical backgrounds (Suk, 2014).

However, Margiotta (2011) asserted that parents’ musical competence has a weak connection with students’ musical learning attainment and suggested that parents of high-achieving students were evenly divided into musically and nonmusically inclined parents. In other words, regardless of parents’ musical competence, is independent of children’s music learning attainment (Margiotta, 2011). For example, some parents who had more than 4 years of music training rarely supervised children’s practices or attended children’s music lessons (Suk, 2014). However, parents with nonmusic backgrounds listen to their children’s musical practices more often than parents who have musical competence (Suk, 2014).

### Cultural diversity

The definition of “culture” is controversial. Numerous researchers have interpreted “culture” as race, which relates to physical characteristics such as facial features, hair type, and skin color (Suk, 2014). Betancourt and Lopez (1993) defined the term “culture” as “highly variable systems of meanings which are learned and shared by people or an identifiable segment of a population, representing designs and ways of life that are normally transmitted from one generation to another” (p. 630). In this paper, the term “culture” is used to advocate and define the behavioral differences related to racial groups (Betancourt and Lopez, 1993).

The literature implies that culturally diverse parents have different values, behaviors and beliefs toward their children’s learning (Swell and Shah, 1968; McNeal, 1999; Lee and Bowen, 2006; Ho, 2011). For instance, most parents in the U.S., even those from minority groups, view education as a significant part of their children’s lives (Fan and Chen, 2001; Jeynes, 2005; Lee and Bowen, 2006). Findings from previous research indicate that white American parents are more involved in their children’s music learning than their minority counterparts are (Mau, 1997). This may be due to low English abilities and a lack of familiarity with the school environment among immigrant groups (Mau, 1997). However, recent research has asserted that even though white parents value education and assist their children in after-school activities, doing homework and studying for performance tests, their participation rates are slightly lower than those of African American parents (Stevenson et al., 1990; Bardhoshi et al., 2016).

Research has shown that parents in Asia value children’s music learning and have high expectations for their children’s educational achievement (Suk, 2014). For this reason, they communicate frequently with teachers about their children’s music education to support their children in achieving success (Suk, 2014). Furthermore, Asian parents have greater lesson attendance than parents from other cultures, especially in music education (Suk, 2014). They are willing to supervise and even practice together with their children (Jeynes, 2005). However, this paper does not emphasize cultural differences among minority groups in the U.S. but rather focuses on cultural differences between the U.S. and China.

## Purpose and research questions

“Extracurricular activities” include participation in clubs, organizations, or other activities outside class that enhance and extend classroom instruction (Mahoney and Cairns, 1997; Darling et al., 2003). In the present study extracurricular music learning refers to music learning outside school. In recent decades, a growing number of parents in both China and the U.S. have foreseen the benefits of music education and enrolled their children in extracurricular music learning (Ilari, 2018). Nevertheless, cross-cultural studies have explore parenting through the lens of music are often left at the margins. Additionally, a limited number of studies have conducted cross-cultural comparisons of parental values toward children’s music learning. Existing cross-cultural comparative studies have focused on the cross-cultural features of musical parents’ influences from Beijing and Hong Kong (Kong, 2018; Kong, 2023). The current cross-cultural study compared the parental values toward their children’s extracurricular music learning in the cities of Guilin and Tampa. By conducting a multiple-case study, we seek to examine the similarities and differences in parents’ attitudes and behaviors regarding their children’s music learning in these two distinct cultural contexts.

This study is guided by the following research questions:

1. How do parents in Guilin and Tampa perceive and value their children’s music learning?
2. Does the value parents hold for their children’s extracurricular music activities differ across different cultural contexts? If so, how do parents’ values and perceptions of their children’s involvement in extracurricular music activities differ between the contexts of Guilin and Tampa?

## Method

This qualitative study employed a multiple-case study design (Yin, 2018) to investigate parental values regarding their children’s music education in four cases in Tampa and Guilin. After obtaining approval from the Institutional Review Board (IRB), we applied the snowball sampling method (Patton, 2015) to identify participants during the recruitment process. A script was created and subsequently emailed to potential candidates known to the researchers in Tampa and Guilin. In situations where individuals were unable to participate, they were encouraged to pass the script along to other eligible families who met the predetermined criteria. The selection criteria for participants included children aged 5–12 years who resided with both biological parents in households located in the two selected cities. The targeted family must be a one-child family and two-child family in each region. Families who expressed interest in the project were instructed to contact us using the provided contact information within the script.

### Study context

In 1979, China implemented the One Child Policy to manage the growth rate of the population. This policy underwent modifications in 2016, allowing couples to have two children, and was further amended in 2021 to permit up to three children. The implementation of the One Child Policy had a direct impact on family size in China, leading to a prevalent three-member family structure. Within this policy context, a generation of single-child emerged (Feng et al., 2014). While the two-child and three-child policies aim to encourage people to have more than one child and increase the fertility rate, the actual effect is not as significant as policymakers expected. Women often prefer having only one child due to the high cost of rearing children and other reasons, especially in urban areas (Zeng and Hesketh, 2016). Since the new policies were implemented relatively quickly, one-child families and two-child families have remained the typical family sizes in China.

In the U.S., there is no population control policy similar to that of China. Individuals generally have freedom in reproductive matters, with the government primarily influencing population growth through alternative measures such as social welfare and tax policies. The transformation in American family size becomes apparent when comparing data from 1976 to the present. The trend indicates a notable departure from larger families, with the average woman in her early 40s now more likely to have two or one child, as opposed to the three or more children commonly found in the 1970s (Livingston, 2015). Concurrently, the percentage of women with three children has remained relatively stable at change to approximately 20%. While the trend toward smaller family sizes is gradually changing, families with two or more children remain typical. Therefore, to ensure that there was a parallel relationship between families in the two selected regions, one-child families and two-child families were the target families.

### Participants

In this multiple-case study, the primary unit of analysis comprised family units consisting of one child and one parent. Importantly, gender differences among the parent participants were not the main focus of this study. Therefore, in the parent interviews, only one parent from each family was interviewed, and the decision regarding which parent would participate was left to the family. In most cases, the parent was the primary caregiver.

The aims of this study were to gain a deeper understanding of parental values regarding children’s extracurricular music education and to explore potential cultural differences across contexts. To achieve these goals, two families from Guilin, China, and two families from Tampa, United States were recruited to participate in this study. In each context, efforts were made to include one family with a single child and one family with two children. The interviews were conducted with children aged 5–8 years old. However, locating a single-child family in Tampa within this specific age range proved challenging. Consequently, the age criterion was expanded to include children aged 5–12 years old. All participating children were required to be born, raised, and currently living with their biological parents. A comprehensive overview of the background details of the study and the participants is presented in Table 1.

**Table 1:** Demographic information of the participants.

Family	Pseudonym and child’s age	Parent interviewed	Interviewed parent’s occupation	Types of music lessons	Price of each lesson
One-child family in Guilin	Ming (5 and half)	Mother	Elementary school music instructor	Violin	500 RMB (70 USD)
One-child family in Tampa	Kate (8 years old)	Father	College instructor	Percussion (learned piano previously)	50 USD
Two-child family in Guilin	Twins Lulu (8 years old) Yoyo (8 years old)	Mother	Businesswoman (was an artist)	Piano	200 RMB (30 USD)
Two-child family in Tampa	Judy (8 years old) Cathy (14 years old)	Father	Nurse	Piano	50 USD

### Data collection

The first author conducted interviews with both the parents and children. The interview protocol is described in the Appendix. The primary source of data for this study was interviews with parents. To ensure the accuracy of the data, interviews with children were conducted as additional information. This approach aimed to reduce the influence of parents on children’s responses and avoid any performance-based answers from parents. The researcher conducted separate interviews with each parent and child within a single case unit. In the second interview, we observed data saturation, during which the same information and themes were consistently repeated. Consequently, we decided to conclude the data collection process. After the interview audio was transcribed, the transcriptions were sent to the parents for member checking.

The data collection process consisted of two phases. In the first phase, structured interviews were conducted with the parents focusing on instrumental selection, behaviors, intentions, decisions, and self-reflection. The duration of these interviews varied from 20 to 60 min. In the second phase, the researcher conducted semistructured interviews with the children, which lasted approximately 20 min. These interviews centered on the topic of interaction between children and parents during the music practice process and the music learning journey. The children’s interview questions were tailored to their psychological development, and utilized language that was easy for them to comprehend and included open-ended questions. For families located in Tampa, in-person interviews were conducted, while families in China were interviewed through online video calls using the WeChat messaging app. This approach allowed for face-to-face conversations in an online setting. The interviews were recorded using a Sony digital recording pen, and the researcher created detailed memos for each interview session.

### Data analysis and validity

The data analysis process comprised two primary phases: single-case analysis and cross-case analysis. Initially, interview memos were collected, and the interviews were recorded and transcribed by the first author. To maintain consistency among the researchers, both the first and second authors coded all the interview transcriptions and examined instances where parents expressed the value of their children’s music education. Each code was labeled and marked based on its frequency and occurrence within the data until saturation was achieved. The coding process followed an inductive approach in which codes were generated and subsequently categorized according to their relevance to the research questions. This systematic process resulted in the identification of five major themes that held significance for the parents’ values. Since this study is a comparative multiple case study, the results followed a comparative structure (Yin, 2018). Based on the cross-case analysis, the researchers created a table (see Table 2) that indicates the similarities and differences between parents from the two cultural contexts across each theme.

**Table 2:** Summary of the results between Guilin and Tampa.

	Guilin parents	Tampa parents	Common issues
Motivation for music learning	1. Children’s autonomy 2. Peer pressures	1. Children’s autonomy 2. Stress relief	1. Children’s autonomy
Expectation of music learning	1. Musical joy 2. “Beauty” development 3. Achievements	1. Musical joy 2. Nonmusical outcomes	1. Musical joy
Utilizing personal background	1. Pursuit of musical career 2. Arts background	1. Pursuit of musical career 2. Integrated human beings;	1. Pursuit of musical career
Strategies for success	1. Teacher selection 2. Extraordinary financial and time investments 3. Practicing 4. Self-reflection	1. Teacher selection 2. Financial and time investments 3. Practicing	1. Teacher selection 2. Financial and time investments 3. Practicing

In a research study involving children, one of the challenges was interpreting children’s poetic and imaginative thoughts. To ensure the validity of this study, the member-checking method was employed (Yin, 2018). We shared the transcriptions with the participants for clarification and to validate the accuracy of the information that was presented. Given the inherent ambiguity in the participants’ understanding and thoughts, we attempted to present a detailed rather than simplistic report. To fully understand the participants’ perspectives, we used direct quotations from the parents and children who maintained their original words without language or grammar corrections (Clark, 2011).

## Findings

The findings are presented according to the four themes that emerged from the data across the four families: (a) motivation for music learning, (b) expectation of music learning, (c) utilizing personal background, and (d) strategies for success (see Table 2).

### Motivations for music learning

Interview data regarding the parents’ motivation to enroll their children in extracurricular music lessons were most helpful for identifying the parental values for children’s music learning. Among them, children’s autonomy was the primary factor that motivated parents from both regions. However, parents from Guilin were influenced by child’s peers to some degree, while parents from Tampa anticipated that their children could relieve stress from life and academic learning through extracurricular music learning.

#### Parents from Guilin

The data were transcribed and coded into two subthemes with the purpose of identifying Guilin parents’ motivations: (a) children’s autonomy and (b) peer pressure.

##### Children’s autonomy

Children’s autonomy in music was the prioritized value that motivated and inspired parents to begin their children’s music lessons, indicating that these parents from Guilin respected their children and encouraged them to pursue their personal interests. In other words, parents ensured that their children maintained ownership and autonomy in the music learning experience. Through interviews with parents and children from Guilin, we confirmed that children were allowed to autonomously select the instrument they wanted to learn. For instance, when we interviewed Ming’s mother about instrumental selection, she explained:

I have a piano at home because I was a music student before. However, Ming preferred the violin rather than the piano, and I saw her passion and talent on this instrument, so we decided to choose the violin for her.

Ming confirmed what her mother said and added:

My aunt sent me a toy violin as a gift. I loved that toy and played with that frequently. After I broke three toy violins, my mom decided to buy me a real violin because she saw that I was passionate about it. I started to learn it at that time.

Yoyo’s mother elucidated that every evening when she hung out with her children, they used to pass through a piano studio, which was located in their living community. Yoyo used to stop by the studio and watch the kids through the vitrine taking piano lessons. When Yoyo expressed her desire to learn about the piano, her mother readily agreed without hesitation.

##### Peer pressures

Another motivator for Chinese parents to enroll their children in music lessons was peer pressure. Parents felt compelled to support their children’s music education after witnessing their friends’ or colleagues’ children participating in private music lessons. These parents were conscious that if they did not provide opportunities for their children, they might lag behind other children of the same age. In essence, they choose to prioritize music education to ensure their children’s competitiveness and future success. Ming’s mother decided to encourage her daughter to develop a hobby after observing her friend’s child taking music lessons. Similarly, Yoyo’s mother supported this idea after witnessing Yoyo’s cousins pursuing music instrument learning.

#### Parents from Tampa

The data from the Tampa parents were also coded and grouped into two subcategories: (a) children’s autonomy and (b) stress relief.

##### Children’s autonomy

Similar to Guilin parents, when Kate’s father was asked the same question regarding motivation to tell his child to learn music, he reinforced the idea of respecting his child’s personal preference:

Her older sister is learning the violin, but she told us that she wanted to try the piano. We have an old piano at home, so she started taking it.

Kate supported her father’s statement with a nod. The abovementioned reasons demonstrated that the children chose their preferred instruments autonomously.

#### Stress relief

Parents from Tampa stated that stress relief was another reason that motivated them to enroll their children in music lessons. Both Judy’s and Kate’s fathers expressed this sentiment, acknowledging the positive impact of music on their children’s well-being. As Judy’s father claimed:

Children have a lot of stress because they must work hard in their academic learning in terms of getting grades and entering a good college. I hope she can transfer her attention from academics and relieve stress through music learning.

Agreed by Kate’s father, who also anticipated his daughter to stay away from academic stresses temporarily, and he believed that music learning might be a good way to relieve stress. In conclusion, children’s autonomy played a dominant role in their enrollment in music lessons in both regions.

### Expectations of music learning

Parents’ expectations partly reflect what they value and how they act toward and how their children learn music. Evidence from the data suggested that musical joy is the primary expectation for music learning which was confirmed by parents from both cities. Guilin’ parents subsequently expected their children to develop their temperament and acquire outstanding achievements, yet parents from Tampa anticipated nonmusical outcomes throughout the learning process.

#### Parents from Guilin

Interview data from Guilin were transcribed and coded into three subthemes: (a) musical joy, (b) “beauty” development, and (c) achievements of Guilin’s parents. The results are presented in the order of importance.

##### Musical joy

Guilin parents held the expectation that their children would experience musical joy on their learning. As Ming mother stated:

She (Ming) enjoys the process of learning the violin, although sometimes it’s not easy to deal with some technique issue, but overall, she feel happy throughout the whole process, which further shaped her characters.

Yoyo’s mother agreed with the magic of learning music. Yoyo’s personality is more extroverted than her sibling. Her mother attributed her extrovertness to the joy that she had taken from the fast tempo and beautiful melody of the musical pieces she played.

##### “Beauty” development

Parents from Guilin recognized the significance of “beauty” through their children’s engagement with music and the aesthetic function of music, which is influenced by the traditional Chinese view of education. The traditional Chinese philosophy of education advocates the idea of the equal importance of five ways of life: virtue, wisdom, health, the collective, and beauty (Luo and Guan, 2023). This highlights the crucial role of beauty, aesthetic development, and artistic sensibility in modern Chinese educational philosophy. As the two mothers from Guilin noted,

Music learning assisted my daughter in shaping her temperament because when she performs on an instrument, she is required to look elegant and follow a certain manner. When she saw other musicians play classical instruments on the screen or on the stage during a concert, the musicians used to dress up real fancy, and so did the audiences.” (Ming’s mother)

Because I was majoring in fine arts before, I am concerned about the aesthetic function of learning art. Indeed, the arts have magic that can shape one’s personality. We used to say that someone has the artist’s temperament. I believe my daughter could acquire this magic through music learning. (Yoyo’s mother)

##### Achievements

Alongside “beauty” development, parents in Guilin are proud of their children’s “achievements” from the competition or instrumental grading tests. As Ming’s mother stated,

My daughter won the first place in a violin competition this year. She is the youngest contestant in the competition, and she performed so well. Competition is a good training for psychological status, and I will definitely sign her up for more competitions.

Yoyo’s mother was proud of her accomplishments not only in competitions but also in piano level examinations. She claimed,

She is eight, and now she passed level 6. Last year, she won the second place in a piano competition, and I am satisfied with her performance.

Hence, parents from Guilin held the belief that music education not only enriched their children’s attributes and refined add their behaviors, fostering their artistic sensibility, and the children’s capacity to value and appreciate diverse art forms, but they also believed that music learning increased their children’s sense of achievement.

#### Parents from Tampa

Data were transcribed and coded into two subthemes: (a) musical joy and (b) nonmusical outcomes with a purpose of exploring Tampa parents’ expectations of toward music learning.

##### Musical joy

In alignment with Guilin parents, Tampa parents emphasized the importance of expecting musical joy through extracurricular music learning, a sentiment echoed by Judy’s father. He asserted that:

Musical joy is the most important part within music learning journey because kids have a lot of pressure from life, and they need to do something fun and creative. Other than joy, I don’t have any expectations for her music learning.

Kate’s father also reinforced the significance of musical joy and stated,

I think percussion learning provides her with a lot of fun. I see her practice with many expressions on her face, and I think those expressions are happiness and enjoyment. In addition, her percussion teacher puts musical joy as the primary goal of each lesson. That’s why Kate anticipates going to her percussion lessons.

Like those in Guilin, parents in Tampa, underscore the crucial role of musical joy in their children’s extracurricular music education. In addition to musical joy, Tampa parents identified some nonmusical outcomes that they expect their children to gain throughout the music learning.

##### Nonmusical outcomes

Both parents in Tampa firmly believed that music learning played a complementary role to their children’s academic development and yielded several positive outcomes that benefited their overall performance. They observed various improvements in their children’s abilities, including an enhanced attention span, improved memorization skills, stronger mathematical aptitude, increased creativity, and a more vivid imagination. For instance, Judy’s father noticed a significant improvement in her attention span and focus on academic learning. As for Kate’s father, he highlighted the positive influence of music learning on her mathematical skills as she counted beats and calculated intervals, which improved her sensitivity to numbers. He believed that this would undoubtedly aid her in her math studies.

### Utilizing personal background

Parents have distinctive values due to their diverse backgrounds which further influenced their decision process or behaviors in terms of their children’s music learning. Under this theme, interview data were transcribed and coded into two subthemes for parents from each region: (a) pursuit of a musical career and (b) an arts background for Guilin parents, and (c) pursuit of a musical career, and (d) an integrated human being for Tampa parents.

#### Parents from Guilin

Interviews for Guilin participants were coded and categorized into two aspects: (a) pursuit of musical career and (b) arts background.

##### Pursuit of musical career

Every family is unique, and parents exert different degrees of effort into supporting children’s musical learning through their personal strengths. The participants agreed that it is possible for them to sustain their children to pursue music as a professional career. Specifically, the two mothers from Guilin stated that they absolutely would support their daughters in pursuing music as a career and that they would financially support such a decision. Nevertheless, due to the diverse backgrounds and occupations, the parents in two different cities displayed distinctive perspectives of their value, as elaborated in the ensuing subthemes: *Arts Background*.

##### Arts background

Parents with art-related backgrounds have more advantages in the process of supporting their children’s musical learning journeys. For instance, both Ming’s and Yoyo’s mothers had arts backgrounds and were familiar with the arts. In other words, they have an in-depth understanding of the journey and process of how to advance musicians and what resources they should contribute within the process; thus, they utilized their strengths to serve their children’s music learning and pursuit of a musical career. For instance, Ming’s mother explained:

We bought two violins, one for her and one for me. The larger size one, which is for adult learners, is for me, and the smaller size one, which is for children learners, is for Ming. When Ming is practicing the violin at home, I use the other violin to practice with her. Additionally, because Ming’s violin lesson is on Saturdays, I decided to assist her practices before Tuesday. After Tuesday, I encouraged Ming to practice by herself, and I sat next to her. If she needs it, I will send her to an affiliated music school to learn professional musical skills.

Similar behaviors were adopted by Yoyo’s mother, as she also stated:

I’d love to take them to the art museum, music concert or have them participate in a music competition to immerse them in an arts atmosphere. I know how expensive it is to pursue music as a career, and those behaviors are absolutely meaningful along their music journey.

Consequently, our observations from the interviews indicate that parents with backgrounds in the arts tend to possess a heightened awareness of art exposure and education. These parents are more inclined to actively engage in artistic learning, taking actions such as learning alongside their children and seeking concert and competition opportunities.

#### Parents from Tampa

According to Tampa parents’ personal background, they valued music learning in two dimensions: they reinforced their children’s pursuit of musical careers and they aimed to foster their children as an integrated human beings.

##### Pursuit of musical career

Like Guilin parents, Tampa parents shared the same opinions about being supportive of their children’s pursuit of music professionally. However, Judy’s father illustrated his opinion about the significance of pursuing music as a career on their own. He further elaborated on about his willingness to support his daughter in pursuing any career that she is keen to pursue. However, she needs to pursue her musical career independently.

##### Integrated human beings

Tampa parents with no art background but a strong educational background wanted to support their child in pursuing music professionally. However, they possessed another kind of opinion as they anticipated that their children would pursue music by themselves as they were aware of the difficulties of pursuing music as a career. As Judy’s father stated:

I will support her if she aims to be a professional pianist, but I hope she is not just a pianist but a knowledgeable person who acquires integrated abilities. In addition, if she decides to pursue, she need to pursue on her own.

In summary, parents’ personal backgrounds may influence their prioritized values of their children, and parents with distinctive backgrounds may value various approaches to support their children in pursuing music as their career.

### Strategies for success

Parents vary in defining “success.” For some, outstanding achievement is the standard, while others view it as an ongoing attitude of deriving satisfaction from continuous learning and practice. Regardless of their interpretation, they employ diverse strategies to support their children toward success.

#### Parents from Guilin

Interview data were transcribed and coded into four subthemes for Guilin’s parents: (a) teacher selection, (b) extraordinary financial and time investments, (c) practicing, and (d) self-reflection.

##### Teacher selection

Evidence indicated that Guilin parents are concerned about selecting the right teacher for their children, putting significant effort into finding the “best match” to support their goals. However, the challenge lies in differing individual standards for what makes a good teacher. For example, Ming’s mother preferred a teacher with professional knowledge and technique. She was determined to be a professional and experienced instructor for Ming, regardless of the difficulty or travel distance involved.

##### Extraordinary financial and time investments

Parents consistently confirmed and endorsed the importance of investment, as they believed that financial investment, time investment, and transportation investment are all indispensable components embedded in the process of children’s music learning. Ming’s mother stated:

Every week, we take a 20-minute high-speed train to another city to take violin lessons. Before the day Ming had a violin lesson, I booked the ticket online and printed the ticket to save more time. We are rarely late for lessons. Sometimes we arrive earlier, and the teacher allows us to audit other students’ music lessons, which inspires Ming to keep up with the good work. Moreover, we are rarely absent from violin lessons even on holidays. We would only be absent if an emergency occurred. During each lesson, I sit next to her, take notes for her, and use my phone to record the whole class for her reference during practice at home.

Even though the duration of a 20-min train ride is short, the amount of time spent on the transportation and commuting from home to the destination on the same day is long and tiring. Furthermore, rather than just dropping Ming off and leaving or waiting outside, Ming’s mother used to sit next to her during lessons and work as an “assistant.”

In addition to time, parents invested varying amounts of money in their children’s music education. For instance, Yoyo’s mother, being a businesswoman, spent extra funds on her child’s music learning due to her limited time for supervising music practice. She hired two distinguished music teachers to improve Yoyo’s music learning. The primary one is for instruction, the other one is for supervising her practice. With two teachers’ coaching, Yoyo’s practice became highly effective, and her piano skills progressed. As for Ming’s mother, she further emphasized the importance of teaching quality, which is directly linked to class tuition. Her goal was to earn more money to ensure a high-quality music education and support Ming musical learning.

##### Practicing

Parents, recognize the crucial role of after-class practice in achieving success, and therefor, they employ various strategies to assist their children. For example, Ming’s mother mentioned that her daughter practiced almost daily for at least an hour. In the early stages, she sat beside her and offered assistance, drawing on her solid musical background. Although her involvement has shifted with her daughter’s advancement to a higher-level of study, Ming’s mother continued to provide support in music theory. In line with the above, Yoyo’s mother, aware of the importance of practice but often busy with her business, hired two music teachers: one for lessons and another specifically for Yoyo’s daily practice.

##### Self-reflection

Parents’ values are shaped by their children’s performance, prompting them to reflect and respond accordingly. For example, Ming’s and Yoyo’s mothers both recognize the significance of musical joy in the learning process. However, they also acknowledge the challenging aspect of skill refinement in music education. Ming’s mother expressed this dual perspective through self-reflection. She regularly self-reflects on whether she was pushing Ming too hard and strives to find the optimal balance between joy and practice.

To this end, it is clear that while achieving success, parents in Guilin adopted and prioritized different strategies or values, including choosing a “good” teacher, investing money or time, accompanying with children’s practice, and self-reflection.

#### Parents from Tampa

Interview data were transcribed and coded into three subthemes for Tampa parents: (a) teacher selection, (b) time investment, and (c) practicing. Both points are equally important for Tampa parents as the strategies for achieving success.

##### Teacher selection

Like parents in Guilin, Judy’s father also highlighted the importance of learning from a good teacher. However, his standard regarding to a “good teacher” was that the teacher has integrated ability such as personality and professional techniques:

My daughter’s piano instructor is really good. She is not only good at the piano but also familiar with other instruments such as the violin and the flute. She is very talented and professional in music learning. She inspired Judy’s musical talent. That’s very important during this journey.

He also underlined the importance of the happiness that the teachers provided to his daughter. He said that his daughter laughed and was joyful after learning from the music teacher. Thus, he believed that in selecting a “good teacher,” the primary standard was personality.

##### Financial and time investments

Parents from Tampa agree that financial and time investments are two crucial elements in their children’s music education. Both fathers confirmed that the cost of each lesson, ranging from 50 to 70 dollars on average, is acceptable. They emphasized that if the price becomes too high, they might consider supporting their child in finding alternatives, such as switching to a more affordable teacher. Regardless of the price factor, both fathers described the significant amount of time they invest in their children’s music education. For example, in terms of transportation, it takes them 30 min to drop their children off at their music lessons. During the lessons, they sometimes wait outside for more than an hour or spend enough time shopping at nearby groceries, allowing them to optimize their time effectively.

##### Practicing

Practicing is a crucial aspect emphasized by parents in Tampa, with Judy’s father highlighting its significance. He firmly believes that, regardless of the subject, dedicated practice is essential. Without a commitment to consistent practice, achieving proficiency in any field, including piano learning, becomes challenging. In support of the importance of practicing, Kate’s father expanded on this by explaining that they do not insist on daily practice at a specific time. However, Kate practices a minimum of 3–4 times per week. Typically, they sit beside her, listening attentively. By doing so, he can identify and correct any incorrect notes or beats during her practice.

The Tampa family with two children recognized the significance of practice, but they had more flexibility in their approach. For instance, Judy’s father stated that he sometimes listened to her practice, and sometimes her older sister assisted with her practice. Through the company of her sister, they are getting closer with each other and her older sister serves as another supportive audience within the family.

### Cross-case synthesis

In this section, we present the results of our cross-case comparison. Table 2 below provides a summary of the similarities and differences identified across four themes within the four cases, spanning two regions. Parents in both regions identified children’s autonomy, musical joys, and pursuit of musical careers as part of the parental values toward their children’s extracurricular music learning. However, parents from different cultural backgrounds value these points from different perspectives. For instance, Guilin parents believed that by happily learning music, children’s personalities were shaped through this learning process. However, Tampa parents believe that happiness is obtained by the child as the child takes extracurricular music lessons. Furthermore, although all the parent participants exhibited their willingness to support their child in pursuing music as a career, parents from Guilin shared that they would fully support their children’s pursuits, while Tampa parents asserted that their children need to pursue a musical career on their own.

Parents’ values are considerably influenced by their individual backgrounds, which has a far-reaching impact on their attitudes and behaviors related to their children’s extracurricular music learning. Coincidently, two of the Guilin mothers acquired an artistic background; in other words, these two mothers witnessed the arduous process of being a professional musicians or artists. In this way, they took numerous actions to foster children’s professional development which included teacher selection unlimited financial investment, and valued the opportunities to sign their child for music competitions or take level exams in order to received recognition from an official institution. In contrast, parents with nonmusical backgrounds in Tampa paid more attention to nonmusical achievements. This may include academic achievement and an integrated competence to fulfill and intensify their curriculum vitae, which is beneficial for the college entrance. The discussion section below will elaborate further.

## Discussion

By interviewing both parents and children, this cross-cultural study compared the values parents place on children’s extracurricular musical learning through four cases: two in Guilin, China, and two in Tampa, United States. Findings revealed that some common issues were generated despite differences in the parental values which corresponded to the findings of previous research and prominent music education philosophies (Xie and Leung, 2011; Jarrett and Coba-Rodriguez, 2015; Caro, 2018; Fung, 2018; Ilari, 2018; Kong, 2018, 2023; Tan, 2019).

### RQ1: How do parents in Guilin and Tampa perceive and value their children’s music learning?

Based on the study findings, we concluded that Guilin parents’ values encompassed respect for their children’s interests, cultivation of beauty and artistic sensibility, professional accomplishments, providing unconditional support, and employing diverse strategies to achieve success. Several factors might have contributed to shaping Guilin parents’ values.

#### The ideology of “beauty and aesthetics”

Notably, most Chinese parents hold the traditional Chinese view that music education is not only for earning a living, but its also for pursuing and developing beauty and manners, which is related to the purpose of aesthetic development in China (Xie and Leung, 2011). However, aesthetic development is a broad context that focuses not only on enhancing beauty and shaping manners, but also on enhancing people’s quality of life (Reimer, 2003). This approach places great importance on training students’ spirit of innovation and practical ability with the long-term purpose of fostering the new generation’s ideology, morality, and cultural awareness.

For the two Guilin mothers with arts specialties, their values were most likely influenced by the beauty and aesthetic development to some degree and were attributed to traditional Chinese philosophy. Returning to the essence of traditional perspectives in ancient and modern China, the Confucian concept of “six arts” and Cai Yuanpei’s idea of the “five ways of life” are deeply rooted and heavily influenced by parents’ perspectives (Tan et al., 2021; Luo and Guan, 2023). In Confucianism’s ideology of “six arts” (*礼lǐ, 乐 yuè, 射 shè, 御 yù, 书 shū, 数 shù*),*礼 lǐ,* which is virtue, plays the dominant role; *乐 yuè*, which is music, plays the second order. Cai Yuanpei also posited virtue as the leading aspect of the “five ways of life” and ended with “beauty” as the contemporary philosophy. Through the two giants’ persepctives of the significance of virtue, music and beauty, we understood that Guilin parents desired to foster their children’s sense of beauty as well as shape elegant and exquisite temperaments through music learning. As such, we inferred that Chinese parents firmly believe in and value the importance of virtue and beauty.

#### Values of collectivism

Another explanation is that traditional Chinese culture is a collectivist culture (Brand, 2004; Tan et al., 2021), which is not only the dominat force in the current Chinese society and schooling, but is also part of the Confucian and Cai Yuanpei’s ideology. Our findings revealed that the Chinese parents were most likely influenced by their peers, which motivated them to encourage their children to learn music. With the rapid development of modern society, children suffer from substantial stress and competition in their pursuit of achievement. Influenced by their peers, Chinese parents feel pressured that their children may be “lost at the starting line.” Therefore, upon noticing that their peers’ children are engaged in music lessons, these parents engage in discussions with their own children to gauge their interest in learning music. Nevertheless, prior research has indicated that Chinese children often aim to avoid disappointing their parents (Comeau et al., 2015). Interestingly, our current study highlights that these parents uphold both their children’s autonomy and the influence of peer pressure as significant considerations. These finding were corroborated by their children’s perspectives as well. However, importantly, we refrain from stating this as a conclusive fact; further evidence is required to substantiate this notion.

#### The ideology of utilitarianism

In the study, Tampa parents’ responses emphasized the advantages of music education as a means to achieve nonmusical outcomes. This result reflected the parents’ belief that music serves a purpose beyond its inherent musical qualities and is aligned with the idea of utilitarianism proposed by Mark (1982). For instance, engaging in music allows children to acquire social–emotional (musical joy) and mathematical skills while improving their ability to focus and pay attention. Many parents also regard music learning as a means for children to develop abilities while building skills that will enhance their chances of being admitted to a prestigious university (Ilari, 2018). In addition, in line with the previous literature, both Tampa parents repeatedly emphasized the significance of music not only for providing happiness, but it also function as stress relief, which are the two prioritized requirements for selecting the music teachers. In other words, Tampa parents focused on affective and cognitive responses to their children (Zdzinski, 1996). Previous research has confirmed that parents exhibit significant affective and cognitive responses, especially at the elementary level (Zdzinski, 1996). We inferred that this result can be viewed as additional evidence that in relevant to the ideology of utilitarianism. Tampa parents firmly believe that happiness and less stress are important aspects for children at the elementary level (Zdzinski, 1996). Thus, what they value is based on their children’s psychological and mental status.

#### Values of individualism

Another possible explanation is the culture of individualism, in which individuals view themselves as the basic unit of survival (Hui and Villareal, 1989). US parents frequently underlined the importance of children’s academic outcomes and college entrance rather than the accomplishment of music learning itself (Hui and Villareal, 1989). In other words, these parents focused on the development of their children themselves. In addition, parents concentrated on children’s psychological status, as they were not only concerned with strengthening their children’s happiness and stress relief, but also illustrated that they would not force their children to practice. This evidence reflected that rather than just focusing on outstanding achievements that children needed, Tampa parents focused on stress reduction as they would not stress their children with daily practice, take piano grading tests or sign up for any type of musical competition. Due to the lack of competition or stress-inducing practices among people in the U.S., these parents were not easily to be influenced by their peers: rather, they focused only on their children’s self-growth only (Tang, 1999). Thus, we conclude that the the U.S. parents in our study posit nonmusical outcomes as the dominant role due to their Western values and cultural context.

## RQ2: Does the value parents hold for their children’s extracurricular music activities differ across different cultural contexts? If so, how do parents’ values and perceptions of their children’s involvement in extracurricular music activities differ between the contexts of Guilin and Tampa?

The present study confirmed that parents act differently according to their different cultural contexts. First, influenced by the traditional Chinese cultures, the two Guilin parents were more actively involved and highly valued in their children’s extracurricular music learning when compared to the Tampa parents. Due to Guilin parents’ arts backgrounds, the two mothers understood the value of music itself. Thus, they paid more attention to the elements of music and provided a better music learning atmosphere for their children, including sitting in their music lessons or accompanying their practice, and signing up for professional music competitions or instrumental grading tests (Comeau et al., 2015; Yuan et al., 2021). They believed that by investing more time and increasing the exposures to music activities, children were more likely to devote more time, creativity, and confidence to the learning journey. Whereas the nonmusical parents from Tampa put more effort on nonmusical outcomes such as psychological support or academic achievement. These results constrast with previous research findings, which indicated that there was no difference between cultural groups when parents did not have a musical background (Comeau et al., 2015). Findings from the current study indicated that parents from Guilin were more actively involved in children’s extracurricular music learning than parents from Tampa.

Second, parents in Guilin place a greater value on competitions and tests, expressing enthusiasm by signing up their children for instrumental grading tests as an achievement in music learning. Surprisingly, parents from Tampa have not expressed any expectations for participating in either competitions or instrumental grading tests. Thus, to some degree, the sociocultural environment might be a factor. In China, a competitive environment places a high value on awards and competitions (Bai, 2021). Some school entrance exams even consider results from art and sports competitions as an additional score in the entrance exam, extending from primary school to high school and even at the college level (Bai, 2021). In the United States, the educational system implements a preferential admission policy that awards additional points for participation and success in extracurricular activities such as art and sports competitions at the college entrance level, typically not involving elementary or high school levels. Therefore, to some degree, Chinese students are exposed to a competitive environment in schooling from primary school to college (Bai, 2021). The parental values of Guilin’s parents discussed that the purpose of extracurricular music learning surpasses the mere achievement in music; it extends to the additional values gained through participation in competitions and tests.

Last, Tampa parents provided more affective and cognitive support than did Guilin’s parents (Zdzinski, 1996). Influenced by the ideologies of utilitarianism and individualism, neither the Tampa family neither enrolled their children in competitions nor had plans to subject them to instrumental grading tests. We hypothesized that Tampa parents might experience more negative influences, such as stress and time consumption, rather than benefits. Tampa parents are more focused on their children’s self-growth, putting more effort into supporting them to lead happy and fulfilling lives. Furthermore, parents in Guilin invested a significant amount of time to support their children, even sacrificing their personal time. This could be attributed not only to the mother’s personal values influence of traditional Chinese culture on mothers’ personal values but also to the historical backdrop of China’s one-child policy, which has instigated heightened parental attention and contributed to their children’s education (Xie and Leung, 2011; Feng et al., 2014).

In summary, we found that with varied values, parents demonstrated descrepent behaviors. We speculated that this difference might be due to the cultural diversity that parents possess which results in varied values toward their children’s extracurricular music learning. Again, every parent wants their children’s lives to be easy and well (Baker and Barg, 2019), in this way, they exhibit “best fit” behaviors, attitudes and values to support their children in becoming a better people.

## Implications

This comparative study offers a unique and crucial perspective on the parental values associated with children’s extracurricular music learning in diverse cultural contexts. The implications of this study hold great significance for music educators, parents, and policymakers, as they encompass aspects such as holistic music education, multicultural music education, parent-educator collaboration, and children’s development.

### Holistic music education

Understanding parental values in relation to children’s extracurricular music learning is crucial for educators to better understand the multifaceted nature of students’ musical development, and to effectively engage both parents and students in music education. Armed with this knowledge, music educators can adapt their curriculum design to bridge the gap between in-school and out-of-school music learning experiences. By incorporating music that meets individual students’ musical needs and connecting the music in school with that outside school, educators can promote a more holistic music education that is meaningful to all students.

### Multicultural music education

The insights gained from exploring parental values in Tampa and Guilin allow music educators to grasp the expectations, diverse needs and values held by parents and children in different cultural contexts. This understanding could prepare music educators develop the knowledge necessary to teach diverse students effectively while accounting for their unique cultural backgrounds. By aligning their teaching practices with parental values and considering the cultural diversity of their students, educators could create a meaningful and enriching music education experiences for all students. For instance, when developing lesson plans, individuals may emphasize not only emphasize musical skills and knowledge as the objective, but they can also consider musical culture, “musical joy” and “beauty” aspects, as well as other nonmusical outcomes. Additionally, they may consider tailoring the teaching method to provide opportunities to engage students musically in a joyful way.

### Parent-educator collaboration

Understanding parental values, especially their strategies for success and personal backgrounds, promotes collaboration between parents and music educators. Communication with parents enables them to stay updated and gain a better understanding of their children’s music learning experiences in school. Additionally, music educators could provide suggestions to parents on how to supervise and support children’s musical practice. In turn, parents could observe their child’s learning progress, challenges, and learning styles and share them with music educators. In this way, the collaboration between educators and parents establishes a supportive environment and effective strategies for a children’s musical education. Additionally, recognizing the cultural differences among families allows for the creation of individualized collaboration plans to support individuals’ success in music learning.

### Development of children

The study’s findings reveal differences in parental values and parenting styles concerning the cultivation of children’s autonomy and responsibility in music learning. Gaining insights into diverse parental values from families with different cultural backgrounds could offer open-minded perspectives for parents, helping them adjust their involvement to better facilitate their children’s music learning. For example, children could be encouraged to engage in informal musical activities with their siblings and peers, and autonomy could be provided in selecting repertoires, instruments, and learning styles. Implementing these strategies could not only contribute to children’s musical education but also enhance their social–emotional learning and sense of responsibility.

## Limitations and future research

The four cases included in this study represented middle-class families from two cities each in China and the U.S. It is essential to note that socioeconomic status was not the primary purpose or criterion for participant selection. The limited participation of low-income or low-SES parents in their children’s music learning might have contributed to their absence in this study (Jarrett and Coba-Rodriguez, 2015; Tan, 2019). Consequently, the findings of this multiple case study cannot be generalized to all parental values concerning children’s extracurricular music learning within these specific cultural contexts. Nevertheless, it is valuable to understand the parental values of children’s extracurricular music learning in low-SES families. This understanding can help educators comprehend the marginalized voices. Future research should aim to explore the value of children’s extracurricular music learning among families of different SES. Additionally, there is a need for further investigation into effective teaching strategies for students from various SES backgrounds and cultural contexts to establish a connection between school music education and extracurricular music learning.

## Conclusion

To summarize, this cross-cultural study compared parental values toward their children’s extracurricular music learning in two selected regions: Guilin, China and Tampa, United States. The study revealed that parents from Guilin respected their children’s interests, paid more attention to the cultivation of beauty and artistic sensibility, and provided unconditional support and diverse strategies to achieve success. The values of parents from Tampa were concentrated on students’ musical joy, stress relief, and nonmusical outcomes. In other words, they anticipate that their children could acquire affective and cognitive support in their lives through the music learning process.

The current findings align with prior studies indicating the active involvement of Chinese parents in their children’s education (Comeau et al., 2015; Yuan et al., 2021; Cui, 2023), with Guilin parents exhibiting greater investments than Tampa parents. However, Tampa parents offered more cognitive and affective support than Guilin parents during their children’s extracurricular music learning. Hence, we concluded that parental values varied based upon their cultural beliefs. Regardless of their actions, parents in both contexts expect their children to achieve success in their musical pursuits. Parents, psychologists, and policymakers must comprehend the various needs and values present in different cultural backgrounds. This understanding is crucial not only for fostering children’s development, crafting curricula, and employing effective teaching methods in the field of music education but also in the broader domains of education, psychology, and sociology.

## Data availability statement

The raw data supporting the conclusions of this article will be made available by the authors, without undue reservation.

## Ethics statement

The studies involving humans were approved by the IRB University of South Florida. The studies were conducted in accordance with the local legislation and institutional requirements. Written informed consent for participation in this study was provided by the participants’ legal guardians/next of kin. Written informed consent was obtained from the individual(s) for the publication of any potentially identifiable images or data included in this article.

## Author contributions

CC: Conceptualization, Data curation, Formal analysis, Funding acquisition, Investigation, Resources, Writing – original draft, Writing – review & editing. XX: Investigation, Methodology, Project administration, Supervision, Validation, Writing – original draft, Writing – review & editing.
