# Supplementary material for: A comparison of the parental values of children’s extracurricular music learning in Guilin, China and in Tampa, United States
Source: Front Psychol. 2024 Jan 30;15:1275734. doi: 10.3389/fpsyg.2024.1275734 (PMC10865502; doi:10.3389/fpsyg.2024.1275734)
Supplement: Supplementary file 1 [file Table_1.DOCX]

**Appendix**

Interview Question for children:

1. What is your favorite musical lesson/ or instruments?

你最喜欢的音乐课程或者乐器是什么？

1. How many classes do you take each week related to music?

课后你有上哪些与音乐相关的课程？

1. How long of your each lesson?

请问你的音乐课每节课有多长？

1. Why do you choose this lesson or instrument?

请问你为什么选择学这个乐器或者声乐？

1. Have you often practiced your instruments?

你经常练琴吗？

1. How long do you practice each day?

请问你每天练琴多长时间？

1. Do you practice alone?

谁陪你练琴呢？

1. Do you prefer practice alone or with your parents?

你希望你爸妈陪你练琴吗？

1. Who choose this lesson for you?

谁替你选择了这个乐器呢？

1. Can you describe a happy memory about learning music?

能不能分享一个你学音乐的时候的开心回忆？

1. What do you think you will do with this instrument/ or singing when you grow up?

你认为你长大以后还会继续学习这个乐器吗？

1. Have you dream about being a musician in the future?

你想不想在未来成为一位音乐家？

1. What is music good for?

你认为音乐能带给你什么？

1. Do your parents enjoy you making music?

当你练琴的时候， 你爸妈一般怎么评价你呢

Interview Questions for Parents:

1. In what music programs do your children participate after school?

请问您的小孩课后有参与什么音乐学习呢？

1. Can you describe your purpose of the children’s music learning?

请问您送小孩去学习音乐的目的是什么呢？

1. What benefit do your children have through music learning?

您认为通过音乐学习小孩能从中有什么收获呢？

1. Which one do you think is more important? Musical Skill or musical joy?

您认为音乐的技能学习更重要还是从音乐中获得快乐更重要？

How do you think music can do that?

您认为音乐能够给孩子带来什么呢？

1. Who made the decision to participate or not to participate in music after school?

请问是谁决定孩子是否学音乐的？

1. Who chose the music activities?
2. Why did you choose these specific musical instruments (activity)?

为什么你们选择了这个乐器？

1. How long do your children practice at home?

请问您孩子每天都练琴吗？

- 1. How long per day?

每天练琴多久？

- 1. How many times and how long for each time?

每周练习几次， 每次练习多久？

1. Do they practice alone or do parents accompany them?

他们练琴的时候你们会陪着吗？还是让他们自己练？

what is your role when your child is practicing?

如果陪着练，你一般会做点什么？

What are you doing when your child is practicing?

如果坐在旁边陪着，你一般都做点啥

Why do/don’t you accompany them?

为什么选择让他独立练琴

Why do they practice alone?

Did anything change?

为什么之前陪着练，现在又不陪了呢？

Why you change your decision?

是什么让您改变了主意

1. Which resources did you put in your child’s (children’s) music learning？

你对你小孩的音乐教育都有哪些投入（时间，精力）?

1. How much money do you spent for your children’s music learning?

你在小孩音乐教育方面的花费？

1. What kind of investment do you put into your child’s music learning?

你对你小孩的音乐教育都有哪些投资?

1. Can you tell me more about your musical background?

您能简单介绍一下你是否有音乐背景？

1. What are your expectations of your children?

你对小孩的音乐学习有什么期待呢？

Do you have any plans to cultivate your child to be a musician in the future?

你对小孩未来的音乐学习有什么期待呢？

If your child decided to become a musician in the future, would you support him/her?

如果您的小孩以后想在音乐方面有所发展您会支持吗？

1. How do you think your educational background influence your children’s music learning?

您认为您的教育经历会影响你小孩的音乐学习吗？

1. Have you participated any music activities?

您有参与任何音乐活动吗？

1. How do you think about music in relation to other subjects in school?

你认为音乐学习会对其他学科的学习有影响吗？

1. What makes music so important to your kids?

您认为学习音乐为什么对您的孩子很重要？

Questions for two children family:

1. How about your other child, is she/he learning music or not?

请问您对另一个孩子有什么音乐学习计划吗？

If YES! Is there any difference between them?

请问是相同的计划还是不同的计划？

Based on your first kid, do you have any different ideas or plans for your second kids’ music studying?

请问是哪里有不同？
